# Association between rs174537 *FADS1* polymorphism and immune cell profiles in abdominal and femoral subcutaneous adipose tissue: an exploratory study in adults with obesity

**DOI:** 10.1080/21623945.2021.1888470

**Published:** 2021-02-17

**Authors:** Chenxuan Wang, Jessica Murphy, Kerri Z. Delaney, Natalie Khor, José A. Morais, Michael A. Tsoukas, Dana E. Lowry, David M. Mutch, Sylvia Santosa

**Affiliations:** a Department of Human Health and Nutritional Sciences, University of Guelph, Guelph, Canada; b Department of Health, Kinesiology and Applied Physiology, Concordia University, Montreal, Canada; c Metabolism, Obesity, and Nutrition Lab, PERFORM Centre, Concordia University, Montreal, Canada; d Centre de recherche - Axe maladies chroniques, Centre intégré universitaire de santé et de services sociaux du Nord-de-l'Ile-de-Montréal, Hôpital du Sacré-Coeur de Montréal, Montreal, Canada; e Division of Geriatric Medicine, McGill University Health Centre, Montreal, Canada; f Division of Endocrinology, Department of Medicine, McGill University, Montréal, Canada

**Keywords:** Fatty acid desaturase, macrophage, T cell, white adipose tissue

## Abstract

Fatty acid desaturase 1 (*FADS1*) polymorphisms alter fatty acid content in subcutaneous adipose tissue (SAT); however, existing evidence is limited and conflicting regarding the association between *FADS1* variants and SAT inflammatory status. To advance this area, we conducted an exploratory study to investigate whether the common rs174537 polymorphism in *FADS1* was associated with immune cell profiles in abdominal and femoral SAT in individuals with obesity. *FADS1* gene expression and immune cell profiles in SAT depots were assessed by qPCR and flow cytometry, respectively. Although *FADS1* gene expression was associated with genotype, no associations were observed with immune cell profiles in either depot. Our study provides additional evidence that rs174537 in *FADS1* has minimal impact on inflammatory status in obese SAT.

## Introduction

Chronic low-grade inflammation in adipose tissue (AT) is characteristic of obesity and its related complications, such as type 2 diabetes and atherosclerosis [1]. Evidence supports that dietary fats, in particular n-3 and n-6 polyunsaturated fatty acids (PUFA), influence AT inflammatory status through the regulation of gene expression, eicosanoid production, and numerous signalling pathways [2]. In general, eicosapentaenoic acid (EPA, 20:5 n-3) and docosahexaenoic acid (DHA, 22:6 n-3) are considered anti-inflammatory, whereas arachidonic acid (AA, 20:4 n-6) is considered pro-inflammatory [2]. While EPA, DHA, and AA can be consumed in the diet, these long-chain PUFA are also endogenously synthesized from the precursor fatty acids α-linolenic acid (ALA, 18:3 n-3) and linoleic acid (LA, 18:2 n-6) in a pathway regulated by fatty acid desaturase and elongase enzymes [3]. Changes in the activity of these enzymes can alter cellular PUFA content and could therefore indirectly influence AT inflammation. This is plausible given that supplementing with n-3 PUFA was reported to regulate immune cell proportions and function [4]. For example, n-3 PUFA have been shown to prevent M1 macrophage and promote M2 macrophage polarization [5,6]. Further, a recent study in fatty acid delta-6 desaturase (D6D) global knock-out mice showed that the inability to convert ALA and LA into long-chain PUFA altered the percentage of CD4 + T cells in the spleen [7]. Given that the infiltration of immune cells, such as macrophages, T cells, and natural killer cells activates inflammatory pathways within AT [1,8], we hypothesized that genetic variants known to modulate desaturase enzyme activity may influence AT immune cell proportions.

Delta-5 desaturase (D5D) and D6D enzymes, encoded by the *FADS1* and *FADS2* genes respectively, regulate endogenous PUFA synthesis in AT [3,9,10]. Considerable evidence shows that single nucleotide polymorphisms (SNPs) in *FADS1/2* genes alter desaturase activities and consequently fatty acid profiles in serum, plasma, and AT [11–13]. However, only a limited number of human studies have investigated if SNPs in the *FADS1/2* gene cluster associate with AT inflammatory markers [11,14,15] and results to date are conflicting. Importantly, these past studies primarily assessed AT inflammatory status by measuring inflammatory gene expression [11,15]. This is a notable limitation given that the use of gene expression to predict protein abundance and functional outcomes becomes increasingly more difficult in tissues comprised of multiple cell-types [16]. Recent evidence suggests that genetic variants are associated with differences in infiltrating immune cell profiles in human tissues [17]. However, it is currently unknown if variants in *FADS1/2* genes modify immune cell proportions in AT. To advance this area, we investigated if the commonly studied rs174537 SNP in the *FADS1* gene was associated with immune cell profiles in abdominal or femoral subcutaneous adipose tissue (SAT) from men and women with obesity.

## Methods

### Subject characteristics

Healthy, non-smoking males and females with a BMI > 30 kg/m^2^ were recruited in a hospital setting from Montreal, Quebec, Canada. Participants were sedentary or untrained, weight stable (± 5 kg) for at least 2 months, free of metabolic conditions and not taking medications that could affect fatty acid metabolism. None of the participants used omega-3 fatty acid supplements. After an overnight fast, body composition was assessed using dual-energy X-ray absorptiometry (Lunar Prodigy Advance; GE Healthcare; Madison, WI) with Encore Software (version 14.10; GE Healthcare; Madison, WI, USA). Blood samples were collected and sent to the McGill University Health Centre Clinical Laboratory – Glen Site for analysis of total cholesterol, HDL-cholesterol, glucose and triglyceride on the Beckman Coulter AU5800 system. Women who were pregnant, breast-feeding or post-menopausal were excluded. The study was approved by the University Human Research Ethics Committee of Concordia University, Comité central d’éthique de la recherche, and the Research Ethics Committee of the CIUSSS North-Island of Montreal. All participants provided written informed consent.

## Collection of abdominal and femoral SAT

Paired abdominal and femoral SAT were collected from each participant after an overnight fast [18]. These upper and lower body fat depots were selected due to their known differences in protein expression [19] and adipokine secretion (e.g., interleukin-6) [20]. The incision site was first sterilized then superficially frozen with 2% lidocaine. A solution of 1:10 lidocaine to lactate ringer was then injected subcutaneously, in a fan shape, using a spinal needle. An incision of ~0.5 cm was made and SAT was aspirated using a 12-gauge tri-eye cannula and 10-ml syringe to create negative pressure. SAT samples were temporarily stored in cell culture media for transport to the laboratory (~15-20 minutes after removal).

## Flow cytometry

A detailed description of the flow cytometry protocol used is outlined in Delaney et al. [21]. Briefly, ~1 g of SAT was washed with PBS and all visible blood vessels were removed. Collagenase digestion was used to liberate the stromovascular fraction (SVF) from the adipocyte fraction. The SVF was then lysed to remove any remaining blood cells and purified through a series of washes and filtrations. Isolated cells were stained with CD68, CD206, CD3, CD4, CD8, and CD45RA antibodies (Supplementary Table 1), and analysed using an 8-colour BD FACSVerse (BD Biosciences, San Jose, CA, USA) and FlowJo software version 9.3.2 (Treestar Inc., Ashland, OR, USA). The gating strategy used for immune cell identification is displayed in **Supplementary Figure 1**.

## DNA extraction and genotyping

DNA was extracted from buffy coats using the Qiagen QIAamp DNA blood mini kit (Qiagen, Toronto, Ontario, Canada). DNA quality and quantity were assessed with a Nanodrop 2000 c (Thermoscientific, Wilmington, DE, USA). The rs174537 SNP in *FADS1* was selected for analysis due to its association with SAT fatty acid content and gene expression, as previously reported [11,15]. Genotyping for the rs174537 SNP was performed using a validated TaqMan assay (Assay ID#: C___2269026_10; Life Technologies, CA, USA) on a Bio-Rad CFX96 Real-Time PCR Detection System (Bio-Rad, CA, USA), as previously described [22]. All samples were genotyped in duplicate to confirm call rate accuracy (100%). All participants consented to genetic analyses.

## Gene expression

RNeasy Lipid Tissue Mini Kit (#74,804, Qiagen) was used to isolate total RNA from SAT samples as per manufacturer instructions. The isolated RNA was then reverse transcribed using the QuantiTect Rev. Transcription Kit (#205,311, Qiagen) into complementary DNA (cDNA) as per manufacturer instructions. cDNA was subsequently used for qPCR using a Bio-Rad CFX96 RT-PCR detection system and SSo FAST EvaGreen Supermix (Bio-Rad Laboratories, Mississauga, ON, Canada), as described previously [23]. To measure *FADS1* mRNA levels, all samples were run in triplicate and normalized to the housekeeping gene *RPLP0*. Primers were designed using the online Roche Universal Probe Library and Assay Design Center. Data was analysed using the ΔΔCt method.

## Statistical analyses

Data were analysed for normality using the Shapiro-Wilks test. Differences between genotypes were assessed with the Mann-Whitney U Test in GraphPad Prism Software 8.0.1, and reported as mean ± SEM. As a secondary analysis, linear regression models were constructed to examine the association between rs174537 and logarithmic transformed immune cell proportions, accounting for participant age, sex, and BMI as covariates (JMP 13 Statistical Software, SAS Institute, Cary, NC, USA). Statistical significance was considered at a P < 0.05.

## Results

This exploratory study was conducted in 35 healthy, non-smoking participants (27 females, 8 males) with a mean age of 32.4 ± 1.0 years and a mean BMI of 37.0 ± 1.2 kg/m^2^. Genotyping for rs174537 in *FADS1* identified 14 major GG allele carriers, 18 GT heterozygous carriers, and 3 TT minor allele carriers. The SNP was in Hardy Weinberg equilibrium (P = 0.40). As commonly reported in the literature, GT and TT carriers were combined into a single group, hereon referred to as ‘minor allele carriers’. Therefore, immune cell analyses compared major (GG) versus minor (GT+TT) allele carriers. Anthropometric and bioclinical measurements for the total population, as well as GG and GT+TT groups, are outlined in (Table 1). Although the distribution of men and women between the two groups was dissimilar, no significant differences in participant characteristics were found between genotype groups.

**Table 1.** Anthropometric and bioclinical measurements of all subjects, as well as GG and GT+TT groups

Metabolic profile	All subjects	GG (n = 14)	GT+TT (n = 21)	P value
Sex (M/F)	8/27	0/14	8/13	-
Age	32.43 ± 1.04	33.00 ± 1.94	32.05 ± 1.20	0.66
BMI	36.98 ± 1.24	39.60 ± 2.20	35.24 ± 1.38	0.09
% Body fat	45.07 ± 1.12	47.65 ± 1.63	43.35 ± 1.43	0.06
Glucose (mM)	4.83 ± 0.08	4.75 ± 0.13	4.89 ± 0.10	0.39
Total cholesterol (mM)	4.45 ± 0.12	4.45 ± 0.20	4.45 ± 0.15	1.00
Triglycerides (mM)	1.35 ± 0.12	1.22 ± 0.14	1.44 ± 0.18	0.39
HDL-cholesterol (mM)	1.23 ± 0.04	1.31 ± 0.06	1.18 ± 0.05	0.13
LDL-cholesterol (mM)	2.61 ± 0.11	2.59 ± 0.17	2.62 ± 0.15	0.89
Total cholesterol/HDL-cholesterol	3.78 ± 0.17	3.50 ± 0.21	3.97 ± 0.23	0.17
Non-HDL-cholesterol (mM)	3.23 ± 0.13	3.15 ± 0.21	3.28 ± 10.16	0.62

P-values indicate statistical difference between major (GG) and minor (GT+TT) allele carriers. Data is reported as mean ± standard error of mean (SEM).

**Figure 1.**
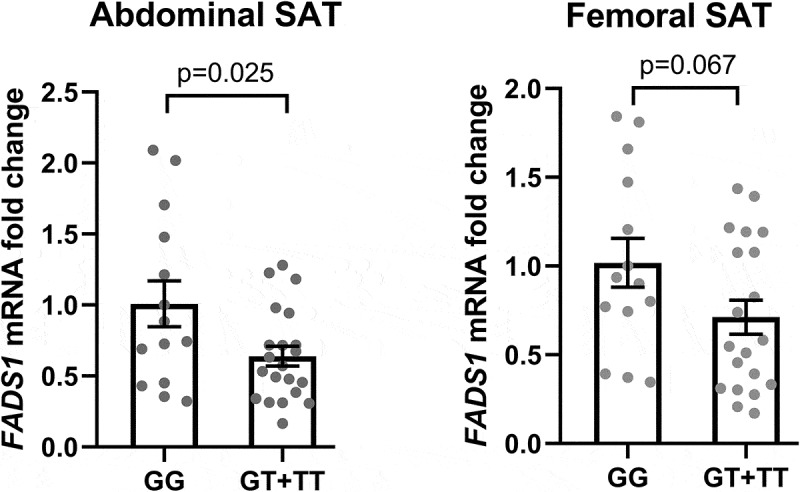
Association between rs174537 and *FADS1* mRNA level in abdominal and femoral subcutaneous adipose tissue (SAT). Individuals were grouped according to rs174537 genotype GG (major) vs. GT+TT (minor). Values are expressed as mean ± SEM

Prior to analysing immune cell proportions, we first confirmed genotype differences in SAT *FADS1* gene expression between major and minor allele carriers. (Figure 1) shows that abdominal SAT from minor allele carriers had lower *FADS1* mRNA levels than major allele carriers (p = 0.025) as expected, while a similar trend was observed in femoral SAT (p = 0.067). We next examined immune cell proportions in participants stratified by genotype.

(Tables 2 and 3) summarize the results in which different immune cell proportions, and related ratios, were compared between major and minor allele carriers in abdominal and femoral SAT, respectively. No differences were found between major and minor allele carriers in either SAT depot for pan T cells (CD3+) or pan macrophages (CD68+), or specific subsets of these immune cells including T helper cells (CD3+ CD4+), cytotoxic T cells (CD3+ CD8+), M2-like macrophages (CD68+ CD206+) and M1-like macrophages (CD68+ CD206-).

**Table 2.** Percentages and ratios of immune cells in abdominal subcutaneous adipose tissue

Immune cell type	All subjects	GG (n = 14)	GT+TT (n = 21)	P value
**% of total live cells**				
CD3+	6.23 ± 0.58	5.17 ± 0.60	6.94 ± 0.86	0.21
CD3+ CD4+ (T helper cells)	2.30 ± 0.26	1.98 ± 0.26	2.52 ± 0.41	0.77
CD3+ CD4+ CD45RA+	0.55 ± 0.10	0.47 ± 0.13	0.61 ± 0.14	0.77
CD3+ CD4+ CD45RA-	1.75 ± 0.19	1.51 ± 0.19	1.91 ± 0.30	0.69
CD3+ CD8+ (cytotoxic T cells)	2.42 ± 0.27	1.81 ± 0.25	2.85 ± 0.40	0.10
CD3+ CD8+ CD45RA+	1.05 ± 0.14	0.76 ± 0.11	1.25 ± 0.21	0.20
CD3+ CD8+ CD45RA-	1.38 ± 0.16	1.06 ± 0.19	1.60 ± 0.24	0.14
CD68+ CD206- (M1-like macrophage)	7.43 ± 1.25	6.15 ± 1.13	8.28 ± 1.94	0.96
CD68+ CD206+ (M2-like macrophage)	3.10 ± 0.60	3.63 ± 1.23	2.74 ± 0.58	0.75
**% of pan T cells (CD3+)**				
CD3+ CD4+	36.57 ± 2.31	39.88 ± 3.46	34.26 ± 3.06	0.34
CD3+ CD4+ CD45RA+	8.10 ± 1.07	8.59 ± 1.65	7.75 ± 1.43	0.59
CD3+ CD4+ CD45RA-	28.48 ± 1.82	31.30 ± 3.20	26.50 ± 2.10	0.32
CD3+ CD8+	38.22 ± 1.96	34.87 ± 2.89	40.57 ± 2.57	0.27
CD3+ CD8+ CD45RA+	16.41 ± 1.45	15.47 ± 1.74	17.07 ± 2.16	0.62
CD3+ CD8+ CD45RA-	21.81 ± 1.79	19.40 ± 2.70	23.50 ± 2.37	0.34
**Ratios**				
CD3+ CD4+ CD45RA-/CD3+ CD4+ CD45RA+	5.68 ± 0.83	4.86 ± 1.12	6.25 ± 1.18	0.29
CD3+ CD8+ CD45RA-/CD3+ CD8+ CD45RA+	1.86 ± 0.32	1.66 ± 0.39	2.00 ± 0.49	0.63
CD68+ CD206-/CD68+ CD206+	7.75 ± 1.98	7.37 ± 2.45	8.00 ± 2.91	0.91

P-values indicate statistical difference between major (GG) and minor (GT+TT) allele carriers. Data is reported as mean ± standard error of mean (SEM).

**Table 3.** Percentages and ratios of immune cells in femoral subcutaneous adipose tissue

Immune cell type	All subjects	GG (n = 14)	GT+TT (n = 21)	P value
% of total live cells				
CD3+	7.38 ± 0.78	6.35 ± 1.16	8.07 ± 1.04	0.28
CD3+ CD4+ (T helper cells)	2.86 ± 0.39	2.45 ± 0.48	3.14 ± 0.57	0.74
CD3+ CD4+ CD45RA+	0.76 ± 0.14	0.67 ± 0.20	0.83 ± 0.20	0.72
CD3+ CD4+ CD45RA-	2.09 ± 0.28	1.78 ± 0.35	2.31 ± 0.40	0.55
CD3+ CD8+ (cytotoxic T cells)	2.89 ± 0.32	2.39 ± 0.49	3.27 ± 0.41	0.27
CD3+ CD8+ CD45RA+	1.28 ± 0.17	0.92 ± 0.22	1.55 ± 0.23	0.09
CD3+ CD8+ CD45RA-	1.61 ± 0.22	1.47 ± 0.37	1.72 ± 0.28	0.46
CD68+ CD206- (M1-like macrophage)	6.00 ± 1.21	5.38 ± 1.55	6.41 ± 1.75	0.75
CD68+ CD206+ (M2-like macrophage)	2.93 ± 0.73	3.28 ± 1.24	2.70 ± 0.91	0.88
**% of pan T cells (CD3+)**				
CD3+ CD4+	38.37 ± 2.30	41.25 ± 3.44	36.36 ± 3.08	0.46
CD3+ CD4+ CD45RA+	10.25 ± 1.18	11.06 ± 1.77	9.68 ± 1.60	0.50
CD3+ CD4+ CD45RA-	28.12 ± 1.78	30.18 ± 3.21	26.68 ± 2.03	0.69
CD3+ CD8+	38.21 ± 2.09	36.20 ± 3.21	39.68 ± 2.78	0.30
CD3+ CD8+ CD45RA+	17.52 ± 1.48	15.93 ± 2.20	18.70 ± 2.01	0.46
CD3+ CD8+ CD45RA-	20.68 ± 1.96	20.28 ± 3.43	20.98 ± 2.37	0.76
**Ratios**				
CD3+ CD4+ CD45RA-/CD3+ CD4+ CD45RA+	5.36 ± 1.22	5.78 ± 2.46	5.04 ± 1.18	0.63
CD3+ CD8+ CD45RA-/CD3+ CD8+ CD45RA+	2.81 ± 1.25	4.36 ± 2.90	1.68 ± 0.42	0.99
CD68+ CD206-/CD68+ CD206+	10.36 ± 3.76	10.34 ± 6.56	10.37 ± 4.62	0.68

P-values indicate statistical difference between major (GG) and minor (GT+TT) allele carriers. Data is reported as mean ± standard error of mean (SEM).

Next, T helper cells (CD3+ CD4+) and cytotoxic T cells (CD3+ CD8+) as a proportion of pan T cells were examined in both their active (CD45RA-) and naïve (CD45RA+) states. No differences were found between major and minor allele carriers for any of these specific T cell-types in either SAT depot. The ratios of active/naïve T helper cells (CD3+ CD4+ CD45RA-/CD3+ CD4+ CD45RA+), active/naïve cytotoxic T cells (CD3+ CD8+ CD45RA-/CD3+ CD8+ CD45RA+) and M1/M2-like macrophages (CD68+ CD206-/CD68+ CD206+) were also examined; however, no genotype-related differences were found in either SAT depot.

A secondary analysis in which we accounted for common covariates such as age, biological sex, and BMI in linear regression models also failed to identify any differences in immune cell types between major and minor allele carriers.

## Discussion

In this exploratory study, we report that the rs174537 SNP in *FADS1* is not associated with immune cell proportions in abdominal and femoral SAT depots in people with obesity despite showing the expected genotype differences in *FADS1* gene expression. We found no differences between GG and GT+TT allele carriers in either SAT depot for any immune cells measured. Additionally, no differences were observed between the two groups regarding the ratios of active/naïve T helper cells, active/naïve cytotoxic T cells and M1/M2-like macrophages. Given the equivocal reports regarding the association between SNPs in the *FADS1/2* gene cluster and AT inflammatory gene expression [11,15], this study provides evidence suggesting that the rs174537 SNP does not influence immune cell proportions in two SAT depots.

PUFA are important regulators of immune function [4]. Evidence suggests that supplementation with long-chain n-3 PUFA can affect immune cell profiles and mitigate inflammation in AT [24,25]; however, translating these past findings to the current study warrant caution. These past studies used cell culture models and mice to investigate the effects of supplemented n-3 PUFA on AT immune cell function. In contrast, the present study did not examine the effects of n-3 PUFA supplementation in humans, but instead focused specifically on the relationship between the rs174537 SNP and immune cell proportions in SAT. Consequently, we can anticipate that the difference in SAT fatty acid profiles following n-3 PUFA supplementation would be considerably greater than that related to a single SNP. Nevertheless, the rs174537 SNP in *FADS1* has been previously shown to modify SAT fatty acid composition. However, it is notable that rs174537 was predominantly associated with changes in SAT n-6 PUFA rather than n-3 PUFA [11]. Irrespective, the association between rs174537 and immune cell populations was unexplored to date. This gap in knowledge was important to address given that changes in immune cell proportions in AT are a key determinant of tissue function.

Hypertrophic adipocytes release several chemoattractant proteins (e.g. MCP-1, CCL5) to recruit various immune cell types into AT [26]. Subsequently, infiltrating immune cells further promote AT inflammation by favouring the production and secretion of pro-inflammatory cytokines (e.g. TNF-α, IL-1β) [26]. Macrophages (M1-like and M2-like) are the most abundant immune cell type in AT. M2-like macrophages are predominantly found in healthy lean AT that express anti-inflammatory cytokines, whereas M1-like macrophages are more abundant in obese and inflammatory AT that produce pro-inflammatory cytokines [1]. Furthermore, T cells also have critical roles regulating AT inflammation. The two major subsets of T cells are CD4 + T helper cells and CD8+ cytotoxic T cells, which secrete cytokines (e.g., TNF-α, IL-2, IL-12 and IFN-γ) that directly impact AT inflammation and promote M1-like macrophage polarization [27]. The present study examined both the types and proportions of the aforementioned immune cells that reside in SAT and revealed that the rs174537 polymorphism in *FADS1* did not associate with any of these immune cell-types in two distinct SAT depots.

The relationship between polymorphisms in the *FADS1/2* gene cluster and inflammatory markers requires additional investigation due to limited and contradictory findings in the literature. For example, Hester *et al*. reported that the rs174537 SNP was associated with serum arachidonic acid levels and oxylipin production in stimulated whole blood [28]. Vaittinen et al. reported that polymorphisms in both *FADS1* (rs174547) and *FADS2* (rs174616) were associated with the expression of an NF-κB pathway gene panel (but not individual inflammatory genes) in obese abdominal SAT after gastric bypass surgery, but not before [15]. Notably, the rs174547 SNP examined in this past study is in high linkage disequilibrium with the rs174537 SNP investigated in the present study (*r^2^ *= 0.967). In contrast, Klingel et al. reported no associations between *FADS1* genotype (rs174537) and 100+ obesity-related genes linked with inflammation, lipid metabolism, and cell differentiation in men and women with obesity [11]. Similarly, Aslibekyan et al. found that SNPs in the *FADS1/2* gene cluster were associated with SAT fatty acid profiles but not high-sensitivity C-reactive protein [29]. Collectively, these conflicting results point to the continued need to study the relationship between *FADS1/2* polymorphisms and inflammation. In the current study, we did not detect associations between rs174537 in *FADS1* and the percentage of identified immune cells, ratios of M1/M2-like macrophages and active/naïve T cells in either SAT depot.

There are several considerations with this exploratory study. First, we only recruited healthy individuals with obesity in this study; thus, our results cannot be generalized to people with normal weight or people with obesity and metabolic syndromes. Indeed, known differences in adipokine secretion and immune cell infiltration in AT from individuals classified as metabolically unhealthy normal weight and metabolically healthy obesity [30] suggest that additional studies in these clinically and metabolically distinct subgroups are warranted. Second, our sample size was small for a genetic association study, but it was deemed reasonable for this first exploratory investigation given the thorough characterization of immune cell profiles in two SAT depots. Third, the distribution of men and women was not equivalent between the major and minor allele groups. While the synthesis of long-chain n-3 PUFA is known to differ between men and women [31], we previously reported in two distinct cohorts that the association between rs174537 and various fatty acids did not differ in a sex-specific manner [32]. Thus, we did not consider this to be an issue. Fourth, we could not measure SAT fatty acid composition due to limited quantities of tissue; however, the significant and expected genotype differences in *FADS1* gene expression meant we had confidence in our immune cell analysis results. A recent meta-analysis showed that the rs174547 polymorphism (i.e., a SNP in near perfect equilibrium with the rs174537 SNP examined in the present study) was associated with reduced desaturase activity [33], suggesting the need to measure both *FADS1* gene expression and fatty acid profiles is redundant for the purposes of this study. Fifth, dietary records were not collected from participants; thus, details regarding dietary fat intake are lacking. However, we did collect information about dietary supplement use and none of the participants used omega-3 supplements. Finally, we only assayed the rs174537 SNP; however, this SNP was documented to have the strongest association with PUFA in genome-wide association studies [34], and its association with AT fatty acid composition has been previously reported [11]. Thus, the need to assay other SNPs was deemed unnecessary.

In conclusion, our exploratory study suggests that the rs174537 polymorphism is not associated with immune cell proportions in SAT depots from healthy, obese individuals. These results contribute to our understanding of the functional outcomes associated with *FADS1/2* SNPs but highlight that continued investigation into the interaction between PUFA, genetic variants and AT inflammation is needed in clinically and metabolically distinct subgroups of individuals.

## Supplementary Material

Supplemental Material
