# Supplementary material for: Association between rs174537 FADS1 polymorphism and immune cell profiles in abdominal and femoral subcutaneous adipose tissue: an exploratory study in adults with obesity
Source: Adipocyte. 2021 Feb 17;10(1):124–30. doi: 10.1080/21623945.2021.1888470 (PMC7894460; doi:10.1080/21623945.2021.1888470)
Supplement: Supplemental Material [file KADI_A_1888470_SM3545.zip › WANG-FADS1andImmuneCells-SuppTable1_final.docx]

**Supplementary Table 1.** Antibody to flourochrome pairings with supplier and clone information and concentration used.

| Antibody | Flourochrome | Supplier | Clone | Antibody Volume  (µl/10^6^ cells) |
| --- | --- | --- | --- | --- |
| CD45RA | FITC | eBioscience | JS-83 | 1.5 |
| CD4 | PE | BioLegend | SK3 | 1.5 |
| CD68 | PE-Cy7 | eBioscience | 815CU17 | 1.5 |
| CD206 | APC | eBioscience | 19.2 | 1.5 |
| CD8 | APC-Cy7 | BioLegend | SK1 | 1.5 |
| CD3 | BV510 | BioLegend | SK7 | 1.5 |
