# Supplementary figures and images for: Association between rs174537 FADS1 polymorphism and immune cell profiles in abdominal and femoral subcutaneous adipose tissue: an exploratory study in adults with obesity
Source: Adipocyte. 2021 Feb 17;10(1):124–30. doi: 10.1080/21623945.2021.1888470 (PMC7894460; doi:10.1080/21623945.2021.1888470)

Supplementary Figure 1

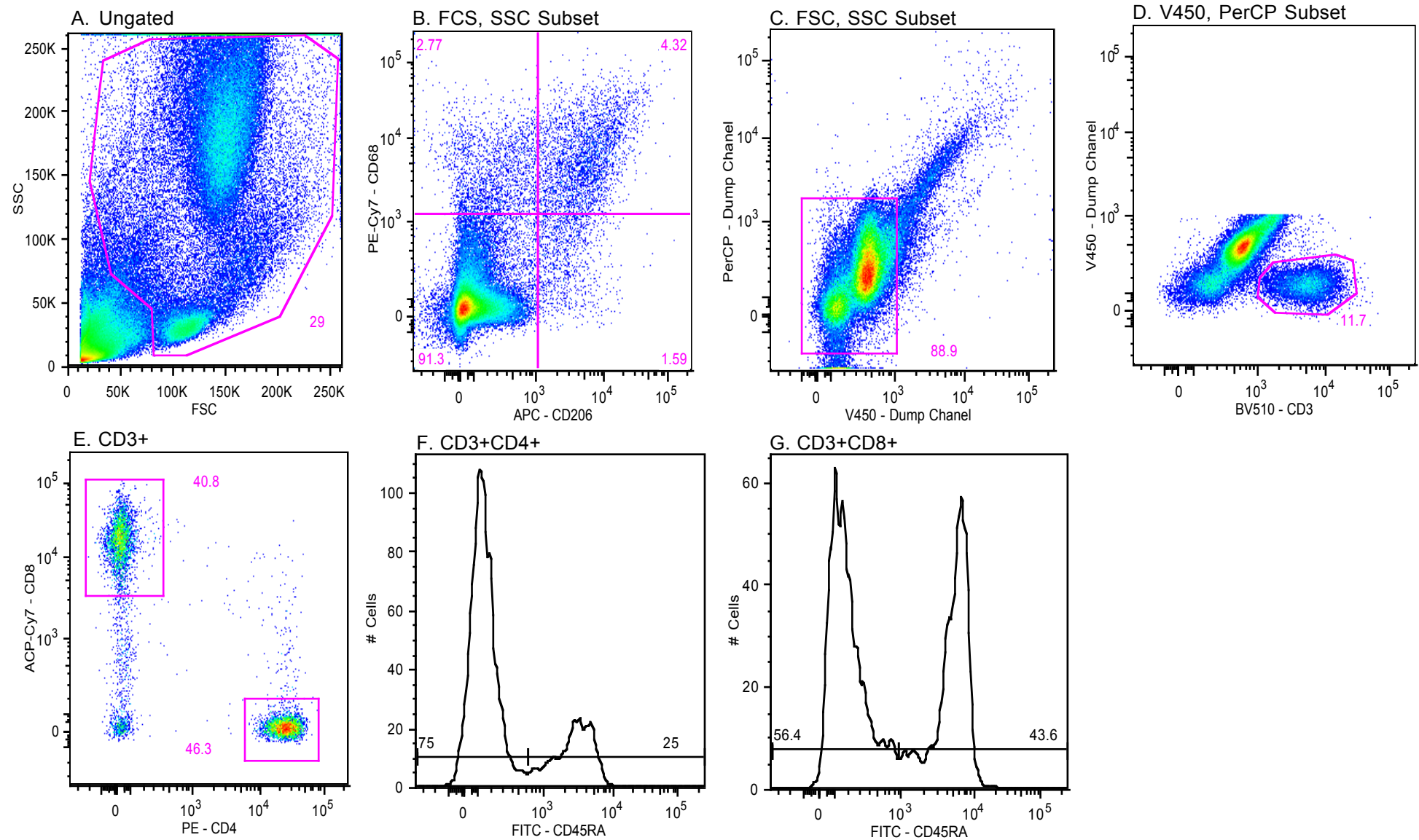

Supplement: Supplemental Material [file KADI_A_1888470_SM3545.zip › WANG-FADS1andImmuneCells-SuppFigure1.pdf]
